# Odor mental imagery in non-experts in odors: a paradox?

**DOI:** 10.3389/fnhum.2013.00087

**Published:** 2013-03-20

**Authors:** Jean-Pierre Royet, Chantal Delon-Martin, Jane Plailly

**Affiliations:** ^1^Olfaction: From Coding to Memory Team, Lyon Neuroscience Research Center, Centre National de la Recherche Scientifique, CNRS UMR 5292, INSERM U1028, University Lyon 1Lyon, France; ^2^INSERM, U836, Equipe Neuroimagerie Fonctionnelle et Perfusion CérébraleGrenoble, France; ^3^Institut des Neurosciences, Université Joseph FourierGrenoble, France

**Keywords:** odor mental imagery, piriform cortex, perfumer, vividness, functional reorganization

## Abstract

In agreement with the theoretical framework stipulating that mental images arise from neural activity in early sensory cortices, the primary olfactory cortex [i.e., the piriform cortex (PC)] is activated when non-olfactory-experts try to generate odor mental images. This finding strongly contrasts with the allegation that it is typically impossible to mentally imagine odors. However, other neurophysiological or cognitive processes engaged in the endeavor of odor mental imagery such as sniffing, attention, expectation, and cross-modal interactions involve the PC and could explain this paradox. To unambiguously study the odor mental imagery, we first argued the need to investigate odor experts who have learned to specifically reactivate olfactory percepts. We then assert the necessity to explore the network dedicated to this function by considering variations in both the activity level and the connection strength of the areas belonging to this network as a function of the level of expertise of the odor experts.

## Is the inability to mentally imagine odors a dogma?

Our ability to mentally imagine visual and auditory scenes or motor actions has been widely demonstrated using behavioral and cerebral imaging studies (Jeannerod, 1995; Halpern and Zatorre, 1999; Kosslyn et al., 2001). However, with regard to olfaction, the widespread assertion is that it is very difficult for the average person to mentally imagine odors. Several authors have even claimed that recalling physically absent odors is not possible (Engen, 1982, 1991; Crowder and Schab, 1995; Herz, 2000), but some behavioral studies tend to take the opposite view (Elmes, 1998; Stevenson and Case, 2005). Two functional imagery studies are in favor of the existence of odor mental imagery because the authors observed activation of the primary olfactory cortex [i.e., the piriform cortex (PC)] in odor-untrained (naïve) subjects (Djordjevic et al., 2005; Bensafi et al., 2007). These results support the general view reported in studies on vision, audition, and motor processes: that similar neural networks are activated during mental imagery and the actual perception of sensory stimuli (Kosslyn et al., 2001).

These findings are nevertheless surprising, as they show that activation of the PC during odor mental imagery is possible in non-experts. Therefore, is the assertion of our inability to mentally imagine odors a dogma? As the Middle Class Gentleman from J. B. Poquelin Molière unknowingly spoke prose, can we unknowingly mentally imagine odors? How can we reconcile the seemingly easy activation of the PC with the apparent difficulty to mentally imagine odors in naïve subjects? Is it possible that the PC activation previously observed in non-experts (Djordjevic et al., 2005; Bensafi et al., 2007) is not associated with an effective mental imagery process? Are we able to improve our capacity for olfactory mental imagination with training? If so, could we observe functional brain modifications associated with these improvements?

## Potential causes of piriform cortex activation

The PC can be activated by other processes than the mental imagery process itself, such as the neurophysiological or cognitive processes engaged in the endeavor of odor mental imagery. First, recent studies have shown that, similar to odor perception, olfactory imagery in naïve subjects is accompanied by increased respiratory amplitude (Bensafi et al., 2003; Kleemann et al., 2009) and that, as a corollary, preventing sniffing during the mental imagery of odor resulted in a poorer image vividness (Arshamian et al., 2008). Because sniffing is not only merely a stimulus carrier but is also a part of the olfactory percept (Mainland and Sobel, 2006), and because sniffing results in PC activation (Sobel et al., 1998; Koritnik et al., 2009), the necessity of the involvement of such an olfactomotor system during olfactory imagery in naïve subjects could explain the PC activation.

Second, activation in the olfactory primary cortex can also be influenced by top-down modulation factors, such as attention or expectation, as subjects are attentive to their olfactory environment while attempting to mentally imagine smell. In agreement with the emerging view of selective attention in the primary sensory processing of vision, audition, and somatosensation in humans (Pugh et al., 1996; Gandhi et al., 1999; Carlsson et al., 2000; Kanwisher and Wojciulik, 2000; Petkov et al., 2004), Zelano et al. (2005) found that subjects were able to pay attention to their olfactory environment while ignoring their auditory environment. Additionally, they found strong attentional modulation at the earliest cortical level of olfactory processing, with the frontal PC responding preferentially to attended sniffs over unattended sniffs. Recently, Zelano et al. (2011) reported that the instructions to prepare for an olfactory task are even sufficient to induce a significant anticipatory response in this region. Rather than a general effect of attention, they highlighted that the activation pattern dedicated to this expectation phase specifically reflects the attended odor. According to the authors, “*the brain generates predictive templates or ‘search images’ in posterior PC, with physical correspondence to odor-specific pattern representations, to augment olfactory perception*.”

Third, PC activation could result from cross-modal associative learning. A visual object previously paired with an unrelated odor, when presented by itself, induces neural activity in the PC in the absence of odor stimulation (Gottfried et al., 2002, 2004). Although evoked after explicit associative learning, this reactivation mechanism is likely similar to the cross-modal activation phenomena observed in the following studies. Reading lips in the absence of any sound or simply reading words with auditory meaning can activate auditory cortices (Calvert et al., 1997; Kiefer et al., 2008). By the same token, food pictures activate gustatory areas (Simmons et al., 2005), and reading words whose meaning have strong olfactory associations automatically activates the PC (Gonzalez et al., 2006); the authors of these studies emphasized that the activation resulted from conceptual reenactments and a process of ignition of the semantic words, respectively, but not from a mental imagery process.

Meyer and Damasio (Damasio and Meyer, 2008; Meyer and Damasio, 2009) proposed a model of cerebral functioning designed to account for how representations are stored in memory so that mental images can be re-experienced during recall. They suggested that “retro-activation uses information available in the association cortices and makes this information explicit by reconstructing maps in the early cortices.” They called these association cortices that enable the multiregional retro-activation of explicit maps in early sensorimotor cortices convergence-divergence zones (CDZs) and suggested that mirror and grandmother neurons operate as CDZs. In olfaction, the orbitofrontal cortex (OFC) and insula are association cortices that could be potential CDZs. We recently showed that observing emotional facial expressions of disgust in others or feeling disgust oneself following odor inhalation activated the same region in the anterior insula (Wicker et al., 2003), suggesting that a mirror-neuron matching system operates for emotional expressions in this region. A cross-modal interaction was also observed at the neuronal level in the monkey OFC with bimodal and even trimodal responses to taste, olfactory, and visual stimuli (Rolls and Baylis, 1994). Furthermore, CDZs can be areas of the motor circuitry, as brain regions involved in understanding others' actions also respond to olfactory cues (Rossi et al., 2008; Tubaldi et al., 2010).

In summary, PC activation can be explained by top-down attentional processes or cross-modal sensorimotor interactions but not necessarily by an odor mental imagery process. This could explain the paradoxical finding that the PC is activated in non-experts who try to mentally imagine a smell, even though their ability to mentally imagine odors is poor to non-existent.

## Vividness of olfactory images

If PC activation is not a decisive criterion to evaluate whether subjects perform odor mental imagery, then what evidence would suggest that such a process is active? In other words, what can be used to determine if vivid olfactory images come into our mind? By evaluating the relationship between the psychophysical evaluation of mental imagery abilities and brain activation, Olivetti Belardinelli et al. (2009) found greater involvement of sensory-specific cortices in high- vs. low-vivid subjects for visual, gustatory, kinesthetic, tactile, and somatic imagery modalities but not auditory or olfactory imagery. They concluded that the vividness scores related to olfactory imagery do not predict olfactory-specific activations, likely due to the difficulty in generating vivid images of smells. This phenomenon could be explained by two particularities of the olfactory system.

First, whereas visual, tactile, and auditory stimuli can be decomposed into multiple components coded in feature maps, such as color, line orientation, movement, or luminous intensity for visual stimuli, odors cannot be decomposed into multiple components. They are induced by chemical molecules, and even the smallest modifications to these molecules can drastically change odor quality. For instance, cis- and trans-p-menth-8-ene stereoisomers have exactly the same molecular formula but are perceived as smelling similar to hydrocarbon or an orange, respectively (Ohloff, 1971). A recent imaging study provided neurobiological evidence that we have a categorical (e.g., woody, minty), not structural (e.g., alcohol, ester), odor quality coding in the posterior PC (Howard et al., 2009). These data support the view that our odor perception is more holistic than analytic, which does not allow for the progressive recall of odor mental images through the gradual gathering of olfactory features and makes the generation of vivid odor images a difficult and even impossible process.

Second, although, as observed for other sensory modalities, olfactory knowledge can be acquired in naïve subjects through perceptual learning by simple prolonged exposure to odors (Li et al., 2006), the difficulty for non-experts to mentally imagine odors clearly differs from other sensory modalities, in which everyone is able to construct conscious vivid mental images and can play the role of an expert. Therefore, the level of olfactory expertise has a strong influence on the ability to generate an odor mental image. Gilbert et al. (1998) found better scores in fragrance experts than non-experts for the vividness of olfactory but not visual images. Studying this specific population is the best method to accurately identify the mental processes underlying the creation of olfactory images.

## Odor mental imagery and expertise level

Perfumers have learned to form olfactory sensory representations through daily practice and extensive training. They claim to be able to produce odor perceptual images in the total absence of odorants. Recently, we took advantage of the variability in expertise level between student and professional perfumers to identify brain areas associated with olfactory mental imagery (Plailly et al., 2012). Briefly, odor names were successively presented. For each name, the experts were asked whether they could mentally imagine the odor as if they physically perceived it. We observed clear differences in the testimonies between groups. Student perfumers reported that odor mental imagery was highly demanding, arduous, and fleeting, despite 2 years of training. A student reported as follows: “*Yes, it requires being really concentrated, it's difficult, really, to see it, to imagine it, to know what it is, it comes as a flash […], it's really hyper-fleeting. It takes time before coming, and when it does happen, it's a fraction of a second.*” By contrast, professional smellers reported that they were able to rapidly evoke odors and maintain mental images for 2–3 s. A renowned perfumer described his experience as follows: “*At the very same time as the word appears, the odor mentally comes out, at the same instant, it's very fast, it's of the second.*” Another reported that “*There are products that I actually use all the time, the image is immediately coming, then this is crazy, because I have realized that raw materials that I use in this moment, information comes straight away, this has stricken me* … ” This outstanding superiority of professionals over student experts, despite a full 2 years of training, highlights the slow development of expertise in perfumers (Schab and Cain, 1992) and reinforces the idea that non-experts cannot mentally imagine odors.

**Figure 1:**
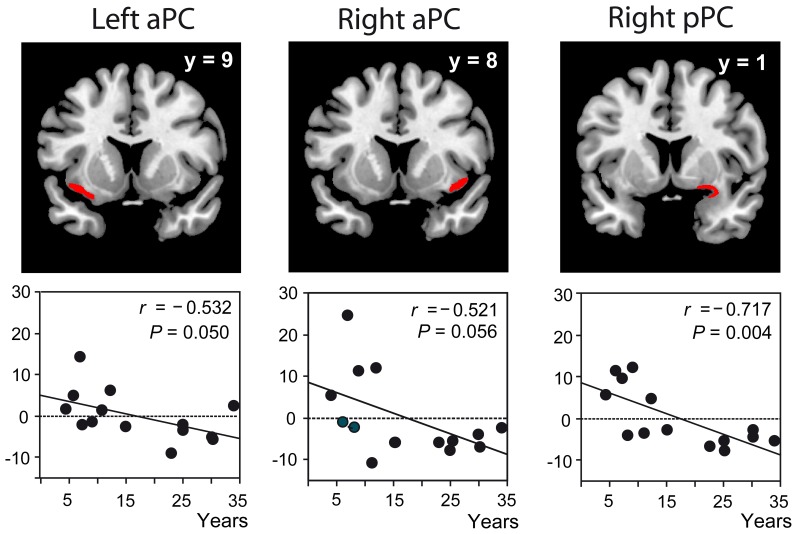
**Functional data in odor experts.** Significant negative correlations between the activation levels (recorded during mental imagery) and length of expertise in the left and right aPC and right pPC represented in red on coronal sections of a normalized, T1-weighted, unsmoothed, structural scan of a professional perfumer. aPC, anterior piriform cortex; pPC, posterior piriform cortex; *y*, coordinate along the antero-posterior axis of the brain; *r*, correlation coefficient; *P*, probability value. Adapted from Plailly et al. (2012).

When professional perfumers generated an olfactory mental image, we observed a signal decrease proportional to the length of expertise in the posterior PC (Figure 1), hippocampus, OFC, and middle frontal gyrus. The greater the level of expertise, the less these regions were activated. The idea of functional reorganization in response to expertise was proposed. We associated this finding with performance gains and high image vividness and emphasized the plasticity of the olfactory system occurring in response to intensive training. The performance gains associated with the activation decrease reveal that brain activations are stronger when the difficulty to perform the task is higher (i.e., in perfumers at the beginning of their career). In the retrieval process taxonomy, this is termed “retrieval effort” and “*refers to the level of processing resources deployed in the service of a retrieval attempt*” (Tulving, 1983).

**Figure 2:**
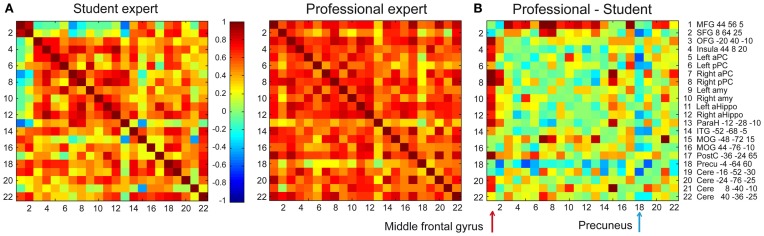
**Coactivation in the odor mental imagery network.** Correlation matrixes depicting functional coactivation between signal time courses of 22 pairs of regions of interest (right column) for olfactory mental imagery events in **(A)** student and professional experts (Plailly et al., 2012). Each cell indicates the group's mean correlation coefficient that was computed between the activation signal of a pair of ROIs. Mean correlation values are shown at the range of −1 (dark blue) to 1 (dark red). The cells depicted in the diagonal of each matrix represent the correlations between the activation level of each region and itself. **(B)** The student coactivation matrix was subtracted from the professional coactivation matrix. This operation shows that the coactivations between the middle frontal gyrus and the rest of the network (red arrows) was higher in professional than student experts (Wilcoxon signed ranks test: *Z* = 3.055, *p* = 0.0022). In contrast, coactivations between the precuneus and other areas (blue arrow) was lower in professional than student experts (*Z* = 2.619, *p* = 0.0088). Abbreviations: MFG, Medial frontal gyrus; SFG, Superior frontopolar gyrus; OFG, Orbitofrontal gyrus; aPC, anterior piriform cortex; pPC, posterior piriform cortex; Amy, amygdala; aHippo, anterior hippocampus; ParaH, parahippocampal gyrus; ITG, Inferior temporal gyrus; MOG, Middle occipital gyrus; PostC, posterior central gyrus; Precu, precuneus; Cere, cerebellum.

In addition to modulating activation levels, performance gains, and high image vividness can be related to changes in odor perceptual coding. First, because odor aversive learning both enhances perceptual discrimination and updates odor quality representations in the posterior PC (Li et al., 2008), we hypothesize that olfactory expertise is similarly associated with a keen discrimination ability and heightened segregation of the odor-specific activity maps. Second, because the retrieval from long-term memory involves the concerted activity of distributed networks (Maguire et al., 2000; Frankland and Bontempi, 2005; Moscovitch et al., 2006), the acquisition of olfactory experience could be associated with an increased strength in the network connections dedicated to the expertise area. Accordingly, when perfumers created odor mental images, the right middle frontal gyrus, a key region in the neural signature of retrieval (Lepage et al., 2000), was strongly coactivated with olfactory and memory regions in professionals; however, in students, this region was not or less coactivated (Figure 2). Because the memory consolidation model called the “multiple trace theory” indicates that the prefrontal cortex plays a crucial role in the posthippocampal recall of remote memories (Frankland and Bontempi, 2005; Takashima et al., 2006), we propose that the middle frontal gyrus could fill this role during the recall of an olfactory percept by ensuring an optimal top-down reactivation of the PC. Another major difference in our study between both groups was the strong coactivation between the precuneus and other regions involved in mental imagery in students, but not in professionals (Figure 2). This disparity makes sense because the precuneus is an area that pertains to the superior parietal lobe, which is active when the need for top-down assistance to memory retrieval is maximal (Ciaramelli et al., 2008).

## Conclusions and future directions

Except for the small minority of individuals whose work leads them to train their olfactory abilities (such as perfumers, chefs, flavorists, and oenologists), most individuals claim not to be able to create an odor mental image, and thus to have the feeling of perceiving a smell in the nose. However, recent experiments take the opposite view and support the existence of odor mental imagery ability in non-experts, using the observation of PC activation during this process as an indicator. We suggest that this paradoxical finding could have several explanations. The PC may be incidentally reactivated during sniffing, odor expectation or attention, cross-modal recall of information previously linked with odor through associative learning, or the ignition of semantic words. Thus, the observation of PC activation in subjects attempting to generate odor mental images does not irrefutably indicate that the odor percept has been reactivated and that the odor has been mentally imagined. Other indices must be taken into account, such as the self-reported ability of subjects to imagine odors, the variations of both the level of activity in olfactory areas and connection strength in the brain network dedicated to this function, depending on the subjects' expertise. By the same token, it would be interesting to study the dynamic of the network involved in generating mental images of odor. For instance, methods exploring effective connectivity, such as dynamic causal modeling (Friston et al., 2003), could be used to test whether the relationship between higher cortical regions and the PC is influenced by the vividness of the olfactory image generated. Furthermore, other topics for future research directions are also conceivable. Howard et al. (2009) have shown that the coding of odor categorical perception is regionally specific for the posterior PC. They used the multivariate techniques that are based on the pattern of voxels activated for a specific stimulus in a specific participant, which allows the characterization of how (rather than just where) the perceptual information is represented in the brain. Using the same multivariate techniques, a challenge for future research would be to investigate the coding of mental images of odor in olfactory experts and answer the following question: is the pattern of activation dedicated to the perception of an odor similar to the one dedicated to the imagination of the same odor? In a similar vein, Howard et al. (2009) have demonstrated that the anterior and posterior PC and OFC contain ensemble representations of individual odorants (odorant identity). However, learning influences odor perceptual coding in the posterior PC only (Li et al., 2008). These same multivariate pattern-based techniques could be used to compare activation patterns between odor experts and naïve subjects to test whether odor percepts are coded with more complex patterns in experts than in naïve subjects and if additional regions, such as the OFC, are involved. Lastly, several studies in humans indicate structural modifications in the brains of musicians and athletes as a consequence of learning and training (Jancke, 2009). Whether there are also structural changes in odor experts remains to be determined. The voxel-based morphometry technique could be used to measure variations in gray and white matter volume in the brain, and the diffusion tensor imaging technique could allow for the exploration of the architecture of white matter and axonal connectivity.

### Conflict of interest statement

The authors declare that the research was conducted in the absence of any commercial or financial relationships that could be construed as a potential conflict of interest.
